# Bluetooth-sensed social presence is associated with immediate vigor and delayed fatigue: A multi-method time series analysis

**DOI:** 10.1016/j.isci.2025.112726

**Published:** 2025-05-22

**Authors:** David Willinger, Stefan Stieger

**Affiliations:** 1 Department of Psychology and Psychodynamics, Karl Landsteiner University of Health Sciences, Krems an der Donau 3500, Austria

**Keywords:** Social sciences, Psychology, Research methodology social sciences

## Abstract

Social interactions affect emotional well-being, yet their temporal dynamics remain understudied in natural settings. We examined these patterns through an experience sampling method study with 80 participants over two weeks (*k* = 3,716 assessments), combining Bluetooth device counts (*n* = 123,574) as a social presence proxy with self-reported emotional states. Using linear mixed-effects models, continuous-time structural equation models, and multi-state Markov analyses, we uncovered complex temporal relationships between social presence and emotions. Our findings revealed that social presence was associated with increased immediate vigor and reduced dejection, followed by delayed fatigue. Bidirectional relationships emerged between social presence and negative mood, with peak effects occurring 3–4 h after initial contact. State-transition analyses demonstrated longer persistence of vigorous states compared to fatigued states, with social presence influencing these transitions. These results demonstrate how social-emotional processes unfold across multiple timescales, suggesting both immediate benefits and delayed costs of social presence.

## Highlights

• Bluetooth device counts serve as valid proxies for social presence in naturalistic settings
• Social presence is associated with immediate positive effects on vigor and delayed fatigue
• Peak effects of social-emotional dynamics occur 3–4 h after initial social contact
• State transitions reveal vigorous states are more stable than fatigued states

## Graphical abstract

**Figure:**
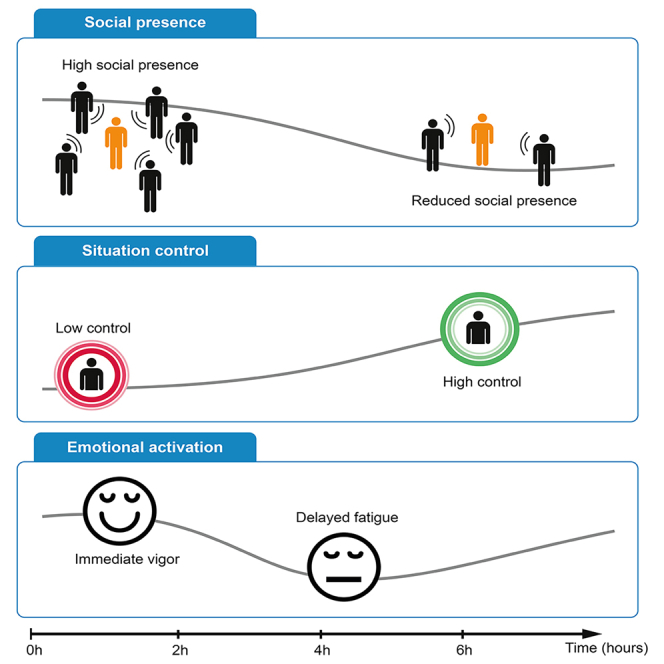

## Introduction

The complex relationship between social interactions and emotional well-being has long been a subject of interest for researchers across various disciplines. Several theoretical frameworks have emerged to explain this relationship: the crowding hypothesis,^1^ social baseline theory,^2^ social buffering,^3^ and social allostasis.^4^^,^^5^ These concepts rely on different aspect human behavior and physiology and thus can offer complementary perspectives on the dynamics relationship of social presence and emotional states. For instance, the stress-buffering hypothesis proposes that social support protects individuals from the harm of stressful events.^3^ Crowding theory extends this by suggesting that high social density can lead to perceived crowding, which then triggers stress responses when individuals lack control over their proximity to others.^1^^,^^6^ Social baseline theory complements the crowding theory by suggesting that the presence of others serves as our default condition for emotional regulation, though this baseline can be disrupted in crowded urban environments with strangers.^7^ The mode of social allostasis proposes that momentary social interactions typically enhance mood and reduce perceived stress (e.g., Sandstrom and Dunn^8^) and that repeated or prolonged social demands may lead to a form of “social allostatic load”.^4^

To study these processes, researchers have increasingly employed experience sampling methodology (ESM) using mobile devices. This approach captures real-time data in naturalistic settings offering high ecological validity.^9^^,^^10^ It has proven particularly valuable in studying dynamic psychological processes which fluctuate throughout the day such as emotions and social interactions.^11^^,^^12^^,^^13^^,^^14^^,^^15^^,^^16^^,^^17^

Recent ESM studies have begun to unravel the temporal dynamics between social interactions and emotional states. Notably, Leikas and Illmarinen^18^ found that extraverted and conscientious behaviors were associated with immediate mood gains but later tiredness. Building on this, Leikas^12^ demonstrated that sociable behavior related to both momentary and future emotional states, particularly fatigue. These findings align with the social allostasis framework, suggesting an initial vigor boost followed by potential depletion of resources. In crowded urban areas, one key mechanism driving this effect might be the invasion of personal space that leads to aversive emotional responses (i.e., crowding theory^19^^,^^20^).

The complex nature of emotional responses to social presence requires careful measurement of distinct affective states. Research has identified four key emotional dimensions particularly relevant to social interactions: vigor, which captures energetic arousal and positive activation; dejection, reflecting negative mood states, and withdrawal tendencies; fatigue, indicating resource depletion; and anger, representing emotional responses to personal space violations.^19^ These dimensions align with theoretical predictions about both immediate and delayed effects of social presence. Recent work in a German-speaking sample has validated reliable measurement approaches for capturing these four distinct emotional dimensions with high temporal sensitivity,^21^ making them particularly suitable for examining dynamic social-emotional processes.

Accurately measuring social environments in daily life, on the other hand, presents methodological challenges. Traditional self-report measures require active input from participants, making frequent or continuous assessment of the social environment impractical and burdensome. This limitation creates substantial gaps in our understanding of how social presence fluctuates throughout the day. To address this need for continuous measurement, researchers have begun to use technology-based solutions that can passively monitor the social environment. Social sensing via Bluetooth device detection has emerged as a promising method for measuring social presence continuously and unobtrusively.^22^ This approach allows for monitoring of nearby devices, serving as a proxy for social presence without relying on participant memory or affecting natural behavior.

To analyze the data generated by this approach, we drew on three complementary statistical methods: linear mixed-effects models (LMMs), continuous-time structural equation modeling (CTSEM), and Markov multi-state models (MSM). Linear mixed-effects models have long been the standard for analyzing ESM data, offering a robust framework for handling nested data structures and accounting for both within- and between-person variability.^23^ However, LMMs operate within a discrete-time framework, which may not fully capture the continuous nature of psychological processes.^24^

To address this limitation, we incorporate CTSEM developed by Driver and colleagues.^25^ This approach extends structural equation modeling to continuous time, allowing for estimation of the rates at which processes unfold and change. CTSEM can provide insights into the temporal order of effects and the speed at which one variable influences another, offering a more nuanced view of social-emotional dynamics. Complementing these approaches, we employ MSM as proposed by Jackson (2011). This method conceptualizes latent states as discrete categories and models transitions between these states as a continuous-time Markov process. Multi-state modeling is particularly useful for studying phenomena involving distinct states or phases, such as mood fluctuations, and can reveal broader macro-level patterns in emotional trajectories influenced by social presence.

The integration of Bluetooth scanning with ESM, combined with multiple analytical approaches, provides a comprehensive examination of socio-emotional dynamics in daily life. First, drawing on research showing that personal space invasions trigger distinct emotional responses^20^ and findings from experience sampling studies on social density,^19^ we hypothesize that social presence, as measured by Bluetooth device detection, will show immediate positive associations with vigor and negative associations with dejection. These responses to social presence may reflect a mechanism for managing social density in urban environments. Second, we expect a delayed increase in fatigue will follow periods of higher social presence, indicating an accumulation of social allostatic load over time. Next, we hypothesize that the temporal dynamics between social presence and emotional states will reveal bidirectional relationships, with peak effects occurring several hours after increased interactions.^12^ Lastly, we will test if distinct emotional states (e.g., vigorous vs. fatigued) will show differential stability influenced by social presence levels. By testing these hypotheses, we aim to explore how our findings on social presence align with and potentially extend aspects of the social allostasis framework, while advancing our understanding of how the broader social environment shapes emotional experiences over time.

## Results

### Sample characteristics and validation of social presence measurements

**Figure 1:**
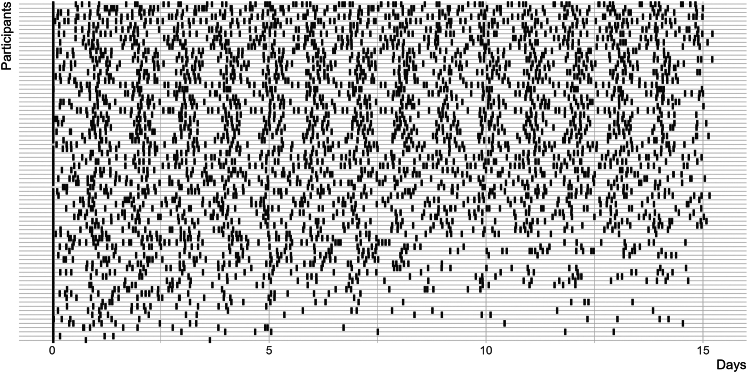
Valid responses across days with continuous spacing of time intervals

The dataset comprised *k* = 3,716 individual datapoints of *n* = 80 participants, of which *k* = 3,542 were complete and retained for further analysis. Throughout the study, participants completed questionnaires that measured four emotional states: vigor, fatigue, dejection, and anger. Each emotional state was assessed through four distinct items rated on a 4-point Likert scale (1 = *not at all*, 4 = *very*), resulting in 16 total items (Table S1). Together with these emotional assessments, participants’ social environment was captured through both self-reported counts of nearby people within their immediate vicinity (2-meter radius), distinguishing between known and unknown individuals, and Bluetooth-based sensing of surrounding devices as proxy for social presence. Individual responses to questionnaires over time are shown in Figure 1. The average self-reported total people count *n*_people counted_ in 2-meter distance at each assessment was *M* = 2.0 (SD = 1.62). The average number of Bluetooth devices *n*_BT_ scanned at each assessment within participants was *M* = 34.1 (SD = 31.6) with an overall median of Mdn = 10. An LMM analysis demonstrated that log*N*_BT_ is a strong predictor of the log-transformed *n*_people counted_ (standardized β = 0.65, 95% confidence interval [CI]: 0.62, 0.68, Figure S1).

### Generalizability analysis demonstrates high reliability of emotional state measurements

The analysis using generalizability theory revealed that the relative variance attributed to differences between participants varied across the four constructs. For scores of fatigue (39.7%), vigor (32.4%), and anger (27.1%) a moderate portion of variance can be attributed to individual differences. Notably, for dejection a higher proportion of 60.5% was attributed to variance between participants, meaning that individual differences are the primary source of variability.

The variance decomposition also showed a notable proportion of variance attributable to the interaction between individuals and the time of measurement for fatigue (32.5%), vigor (34.7%), and anger (36.1%) suggesting that individual variability in these three constructs is substantially varying over time. Dejection had a slightly lower interaction coefficient of 15.1%, indicating that temporal variability is relatively smaller. Items of all constructs were relatively homogeneous as reflected in the small variance component of items (fatigue: 1.8%, vigor: 2.1%, dejection: 0.3%, and anger: 3.9%).

Finally, the reliability as measured by the generalizability coefficient was large for all constructs (ρ^2^_fatigue_ = 0.95, ρ^2^_vigor_ = 0.93, ρ^2^_dejection_ = 0.97, and ρ^2^_anger_ = 0.93), indicating excellent consistency of the emotion state measurements across time and items. These results suggest that a large proportion of the observed score variance can be attributed to true score variance rather than measurement error. In practical terms, this means our instrument reliably captured genuine fluctuations in participants’ emotional states, allowing us to differentiate between individuals and track changes within individuals over time with high confidence. The average scores of the emotional state questionnaire are summarized in Table S1 while all variance components are summarized in Table S2.

### Linear mixed-effects model: Immediate and lagged relationships between sensed social presence and emotion

We analyzed the associations between emotional states and social presence with an LMM including individual assessments (level 1) nested in participants (level 2), with log*N*_BT_ as the dependent variable, and emotional state variables and situation control as the predictors.

The analyses revealed interesting patterns in the relationship between log*N*_BT_ and mood states. In the contemporaneous model, significant within-person effects showed that higher social presence was associated with lower dejection (β_20_ = −0.06, *p* < 0.01), higher vigor (β_40_ = 0.08, *p* < 0.001), and lower situation control (β_50_ = −0.27, *p* < 0.001). These findings suggest that momentary increases in social presence correlate with immediate increases in vigor and decreases in dejection, along with reduced feelings of control.

In the lagged model, examining future mood states, we found a different pattern. Higher social presence predicted lower future dejection (β_20_ = −0.04, *p* < 0.05) and reduced situation control (β_50_ = −0.03, *p* < 0.05), though with small effect sizes. Interestingly, higher social presence showed a positive relationship with subsequent fatigue levels (β_30_ = 0.07, *p* < 0.01). Between-person effects were less pronounced in both models, with only anger approaching significance (contemporaneous: β_01_ = 0.23, *p* = 0.08; lagged: β_01_ = 0.24, *p* = 0.06). This suggests that within-subject heterogeneity plays a crucial role in the relationship of social presence and mood. The models’ explanatory power was comparable and both captured a substantial part of the variance (contemporaneous: *R*^2^_conditional_ = 53%, *R*^2^_marginal_ = 9%; lagged: *R*^2^_conditional_ = 49%, *R*^2^_marginal_ = 5%). The results are summarized in Table 1 for contemporaneous effects and Table 2 for lagged effects.

**Table 1:** Results of the linear mixed-effects model analysis for contemporaneous associations between number of Bluetooth devices and mood states

	Fixed				Random	
	Coeff.	*B* [95% CI ]	β	*t*	Coeff.	*SD*
Intercept	γ_00_	2.43 [2.21, 2.66]	2.45	21.24∗∗∗	u_0*j*_	0.95
Within-person (all.cwc)						
Anger	β_10_	0.00 [−0.02, 0.01]	−0.01	−0.53		
Dejection	β_20_	−0.02 [−0.04, −0.01]	−0.06	−3.31∗∗		
Fatigue	β_30_	−0.01 [−0.02, 0.00]	−0.04	−1.70		
Vigor	β_40_	0.02 [0.01, 0.03]	0.08	4.03∗∗∗		
Situation control	β_50_	−0.02 [−0.02, −0.02]	−0.27	−15.15∗∗∗		
Time	β_60_	−0.24 [−0.62, 0.13]	0.02	−1.27	u_1*j*_	0.03
Between-person (all.cmc)						
Anger	β_01_	0.11 [−0.01, 0.23]	0.23	1.75		
Dejection	β_02_	−0.08 [−0.16, 0.00]	−0.37	−1.96		
Fatigue	β_03_	0.02 [−0.07, 0.11]	0.09	0.71		
Vigor	β_04_	−0.01 [−0.09, 0.07]	−0.03	−0.23		
Situation control	β_05_	−0.01 [−0.03, 0.00]	−0.25	−1.90		

*R*^2^_conditional_ = 53%, *R*^2^_marginal_ = 9%; AIC = 10,064, BIC = 10,169, ICC = 49%, ρ ≈ 2.5·10^−5^. *k* = 3,516 observations nested within 80 participants. cwc = centered within cluster; cmc = centered at the mean of the cluster. ∗*p* < 0.05, ∗∗*p* < 0.01, and ∗∗∗*p* < 0.001.

**Table 2:** Results of the linear mixed-effects model analysis for lagged (*i* + 1) associations between number of Bluetooth devices and future mood states

	Fixed				Random	
	Coeff.	*B* [95% CI]	β	*t*	Coeff.	*SD*
Intercept	γ_00_	2.37 [2.15, 2.59]	2.36	20.97∗∗∗	u_0*j*_	0.93
Within-person (all.cwc)						
Anger	β_10_	0.00 [−0.01, 0.01]	0.00	0.27		
Dejection	β_20_	−0.02 [−0.03, −0.00]	−0.04	−2.22∗		
Fatigue	β_30_	0.02 [0.01, 0.03]	0.07	3.29∗∗		
Vigor	β_40_	0.00 [−0.01, 0.01]	0.00	0.12		
Situation control	β_50_	−0.002 [−0.002, −0.001]	−0.03	−2.25∗		
Time	β_60_	0.01 [−0.00, 0.02]	0.01	1.66	u_1*j*_	0.04
Between-person (all.cmc)						
Anger	β_01_	0.11 [−0.01, 0.23]	0.24	1.87		
Dejection	β_02_	−0.07 [−0.15, 0.01]	−0.31	−1.80		
Fatigue	β_03_	0.02 [−0.08, 0.10]	0.07	0.41		
Vigor	β_04_	−0.01 [−0.08, 0.07]	−0.04	−0.21		
Situation control	β_05_	−0.01 [−0.03, 0.00]	−0.17	−1.49		

*R*^2^_conditional_ = 49%, *R*^2^_marginal_ = 5%; AIC = 10,092, BIC = 10,196, ICC = 47%, ρ ≈ 3.3·10^−5^. *k* = 3,438 observations nested within 80 participants. CI = confidence interval, cwc = centered within cluster; cmc = centered at the mean of the cluster. ∗*p* < 0.05, ∗∗*p* < 0.01, and ∗∗∗*p* < 0.001.

### Convergent validity of social presence measures

To assess the robustness of our Bluetooth-based social sensing approach, we conducted parallel analyses using self-reported counts of people within 2m distance. The LMMs revealed remarkable convergence between both, supporting the validity of Bluetooth device counts as a proxy for social presence.

In the contemporaneous models, both measurement approaches captured consistent patterns of emotional dynamics. Self-reported social presence showed significant associations with reduced dejection (β_20_ = −0.06, *p* < 0.001) and enhanced vigor (β_40_ = 0.09, *p* < 0.001), closely mirroring the Bluetooth-based findings (dejection: β_20_ = −0.06, *p* < 0.01; vigor: β_40_ = 0.08, *p* < 0.001). Both methods also captured decreased situational control with increased social presence, though the effect appeared stronger in Bluetooth measurements (β_50_ = −0.27, *p* < 0.001) compared to self-reports (β_50_ = −0.20, *p* < 0.001).

The temporal dynamics revealed through lagged analyses demonstrated strong similarities. Both measurement approaches identified a delayed fatigue response of similar magnitude (bluetooth: β_30_ = 0.07, *p* < 0.01; self-report: β_30_ = 0.07, *p* < 0.01). Some divergence emerged in other lagged effects: while Bluetooth measures showed persistent sensitivity to dejection (β_20_ = −0.04, *p* < 0.05), self-reported counts maintained associations with vigor (β_40_ = 0.03, *p* < 0.05).

The convergence of fixed effects across social presence measures (Bluetooth and people count), particularly for immediate emotional responses, provides strong evidence for the validity of our technological approach to measuring social proximity. The results for self-reported social presence are summarized in Tables 3 and 4.

**Table 3:** Results of the linear mixed-effects model analysis for contemporaneous associations between number of people counted and mood states

	Fixed				Random	
	Coeff.	*B* (95% CI)	β	*t*	Coeff.	*SD*
Intercept	γ_00_	0.67 [0.59, 0.75]	0.64	16.49∗∗∗	u_0*j*_	0.26
Within-person (all.cwc)						
Anger	β_10_	−0.01 [−0.02, 0.00]	−0.02	−1.55		
Dejection	β_20_	−0.02 [−0.03, −0.01]	−0.06	−4.56∗∗∗		
Fatigue	β_30_	0.00 [−0.01, 0.01]	−0.00	−0.10		
Vigor	β_40_	0.02 [0.01, 0.03]	0.09	5.74∗∗∗		
Situation control	β_50_	−0.01 [−0.02, −0.01]	−0.20	−14.97∗∗∗		
Time	β_60_	−0.00 [−0.01, 0.00]	−0.02	−0.90	u_1*j*_	0.02
Between-person (all.cmc)						
Anger	β_01_	0.03 [−0.01, 0.07]	0.06	1.34		
Dejection	β_02_	−0.03 [−0.06, −0.00]	−0.14	−2.17∗		
Fatigue	β_03_	0.02 [−0.01, 0.05]	0.10	1.57		
Vigor	β_04_	0.02 [−0.01, 0.00]	0.06	1.32		
Situation control	β_05_	−0.00 [−0.01, 0.00]	−0.07	−1.46		

*R*^2^_conditional_ = 49%, *R*^2^_marginal_ = 5%; AIC = 6,834, BIC = 6,937; ICC = 47%, ρ ≈ 3.2·10-7. *k* = 3,190 observations nested within 74 participants. CI = confidence interval, cwc = centered within cluster; cmc = centered at the mean of the cluster. ∗*p* < 0.05, ∗∗*p* < 0.01, and ∗∗∗*p* < 0.001.

**Table 4:** Results of the linear mixed-effects model analysis for lagged (*i* + 1) associations between number of people counted and future mood states

	Fixed				Random	
	Coeff.	*B* [95% CI]	β	*t*	Coeff.	*SD*
Intercept	γ_00_	0.61 [0.52, 0.69]	0.63	14.25∗∗∗	u_0*j*_	0.26
Within-person (all.cwc)						
Anger	β_10_	0.00 [−0.01, 0.01]	0.01	0.55		
Dejection	β_20_	−0.01 [−0.02, 0.00]	−0.02	−1.19		
Fatigue	β_30_	0.02 [0.01, 0.03]	0.07	4.71∗∗		
Vigor	β_40_	0.01 [0.00, 0.02]	0.03	2.28∗		
Situation control	β_50_	−0.00 [−0.00, 0.00]	−0.05	−0.63		
Time	β_60_	0.00 [−0.01, 0.01]	0.01	0.72	u_1*j*_	0.02
Between-person (all.cmc)						
Anger	β_01_	0.03 [−0.01, 0.07]	0.06	1.45		
Dejection	β_02_	−0.07 [−0.05, 0.01]	−0.10	−1.53		
Fatigue	β_03_	0.02 [−0.01, 0.05]	0.08	1.22		
Vigor	β_04_	−0.01 [−0.01, 0.05]	0.07	1.51		
Situation control	β_05_	−0.00 [−0.01, 0.00]	−0.05	−0.96		

*R*^2^_conditional_ = 17%, *R*^2^_marginal_ = 2%; AIC = 6,919, BIC = 7021, ICC = 15%, ρ ≈ 3.3·10^−7^. *k* = 3,118 observations nested within 74 participants. CI = confidence interval, cwc = centered within cluster; cmc = centered at the mean of the cluster. ∗*p* < 0.05, ∗∗*p* < 0.01, and ∗∗∗*p* < 0.001.

### Continuous-time structural equation modeling: Bidirectional dynamics of social presence and emotion

The CTSEM analysis shed light on the dynamics between Bluetooth-sensed social presence and emotional states, offering a more nuanced perspective on the temporal interactions previously hinted at in the lagged LMM. For the factor loadings (Λ) of the negative mood state, fatigue was the strongest indicator (λ = 0.68, 95% CI: 0.42, 0.95), followed closely by vigor with a negative loading (λ = −0.58, 95% CI: -0.82, −0.37). Dejection (λ = 0.18, 95% CI: 0.11, 0.25) and anger (λ = 0.13, 95% CI: 0.07, 0.18) were significantly but not as strong related to negative mood state.

**Figure 2:**
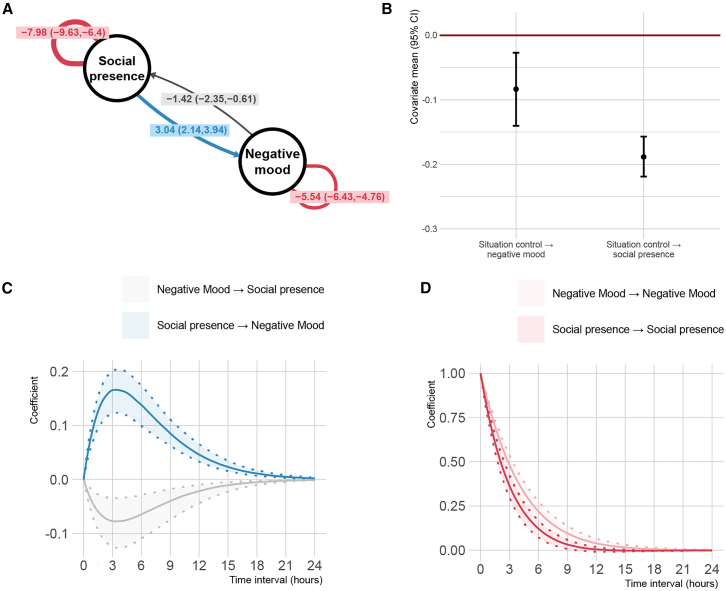
Temporal dynamics of social presence and negative mood revealed by continuous-time structural equation modeling (A) Social presence is having an increasing effect on negative mood, while negative mood has a decreasing effect on social presence. (B) Situation control has an instantaneous negative effect on social presence and negative mood. Parameter values are presented as mean and 95% confidence interval. (C) The lag of the peak effect of the cross-effect social presence on negative mood was 3 h and 7 min, white the lag of the peak effect of negative mood on social presence was 3 h and 50 min. Shaded areas represent the 95% credible intervals. (D) The half-life of the auto-effect of negative mood was 2 h 38 min, while the half-life of social presence was 1 h and 55 min. Shaded areas represent the 95% credible intervals.

Assessment of the drift parameters (*A*) showed an intricate feedback loop between the social presence and mood (Figure 2A). Crucially, we identified a bidirectional relationship, namely that negative mood was negatively associated with social presence (*a* = −1.42, 95% CI: −2.35, −0.61), while a higher social presence was associated with a higher negative mood (*a* = 3.04, 95% CI: 2.14, 3.94). Moreover, both the social presence (*a* = −7.98, 95% CI: −9.63, −6.40) and negative mood (*a* = −5.54, 95% CI: −6.43, −4.76) exhibited strong self-regulation indicated by the negative self-connection (Figure 2A).

*Situation control* was as a significant time-dependent covariate in the model, indicating a modulating effect on both social presence and emotional states (Figure 2B). It exhibited a robust negative association with social presence (*b* = −0.19, 95% CI: −0.22, −0.16) and negative mood state (*b* = −0.08, 95% CI: −0.14, −0.03), implying that as perceived control increases, negative mood and social presence decrease.

The latency of the peak impact of the cross-effect of social presence on negative mood was 3 h and 7 min, while the latency for the peak effect of negative mood influencing social proximity was slightly longer, at 3 h and 50 min (Figure 2C). Negative mood showed a half-life (i.e., time needed to reduce its intensity by 50%) of 2 h and 38 min. In comparison, the half-life of social presence was notably shorter, at 1 h and 55 min (Figure 2D). The complete model results are summarized in Table 5 and shown in Figure 2.

**Table 5:** Estimated parameters of the continuous-time structural equation model

Parameter	Mean	SD	95% CI
Initial states T0			
Social presence (Mean)	−0.18	0.08	[−0.33, −0.03]
Negative mood state (Mean)	0.59	0.18	[0.23, 0.95]
Social presence (Variance)	0.56	0.06	[0.45, 0.67]
Negative mood state (Variance)	1.12	0.26	[0.70, 1.69]
Mood state and social presence covariance	−0.03	0.08	[−0.18, 0.12]
Factor loadings Λ			
Fatigue	0.68	0.13	[0.42, 0.95]
Vigor	−0.58	0.11	[−0.82, −0.37]
Dejection	0.18	0.04	[0.11, 0.25]
Anger	0.13	0.03	[0.07, 0.18]
Drift parameters A			
Social presence → social presence	−7.98	0.82	[−9.63, −6.40]
Negative mood → negative mood	−5.54	0.43	[−6.43, −4.76]
Negative mood → social presence	−1.42	0.44	[−2.35, −0.61]
Social presence → negative mood	3.04	0.47	[2.14, 3.94]
Diffusion parameters G			
Social presence	2.55	0.20	[2.20, 2.95]
Negative mood state	3.01	0.62	[1.99, 4.45]
Cross-diffusion: Mood state and social presence	−0.08	0.02	[-0.12, −0.03]
Time-dependent covariates b			
Situation control → social presence	−0.19	0.02	[−0.22, −0.16]
Situation control → negative mood	−0.08	0.03	[−0.14, −0.03]
Measurement parameters			
Measurement error: Bluetooth log	0.25	0.05	[0.17, 0.36]
Measurement error: fatigue	0.32	0.02	[0.29, 0.36]
Measurement error: vigor	0.55	0.01	[0.53, 0.57]
Measurement error: dejection	0.44	0.01	[0.43, 0.45]
Measurement error: anger	0.76	0.01	[0.74, 0.78]
Manifest mean: Bluetooth log	−0.02	0.08	[−0.19, 0.14]
Manifest mean: fatigue	0.06	0.08	[−0.10, 0.23]
Manifest mean: vigor	−0.03	0.07	[−0.17, 0.12]
Manifest mean: dejection	0.02	0.10	[−0.17, 0.22]
Manifest mean: anger	0.01	0.08	[−0.14, 0.16]

Standardized variables. CI = credible interval. The 95% CI represents the lower and upper bounds of the 95% credible interval.

### Multi-state modeling: Stability and transitions in mood states influenced by social presence

**Figure 3:**
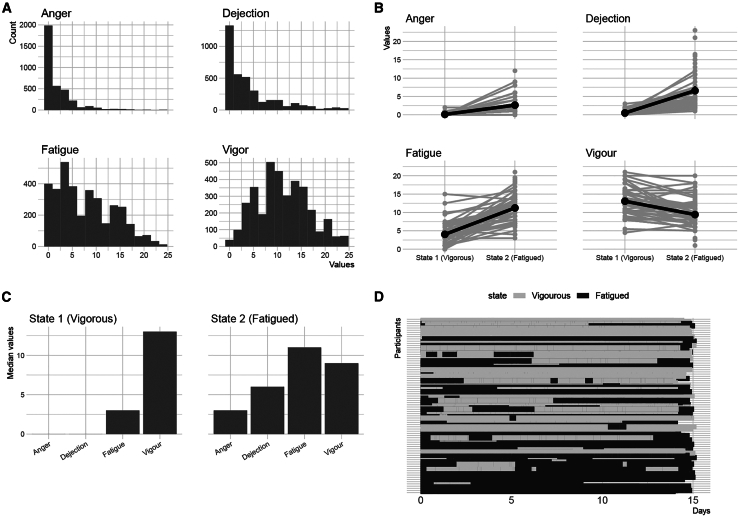
States recovered by the two-state model (A) Distribution of the emotional states, suggesting a zero-inflated negative binomial. (B) Individual recovered emotional states and median (bold) across group. (C) Summary of the emotional state profiles across the group. Shows that state 1 is vigorous, in state 2 fatigue is prevalent. (D) Time courses of vigorous and fatigued state across the time of study.

Lastly, the multi-state model revealed insights into the temporal structure of discrete emotional state fluctuations (Figure 3A), offering an additional view on the macro-level. The model identified two states, which corresponded to a vigorous and a fatigued mood, based on their respective emotional state profile (Figures 3B and 3C). Our analysis examined mean sojourn times (i.e., the average duration before transitioning to a different state) to assess the stability of emotional states. The longer sojourn time for the vigorous state (*M* = 10.72 days, 95% CI: 7.57, 15.17) compared to the fatigued state (*M* = 6.20 days, 95% CI: 4.67, 8.22) suggests that a vigorous emotional state tends to be more self-sustaining (i.e., more and longer; Figure 3D). Conversely, transition probabilities quantify the likelihood of moving between states within a specified time frame—in this case, one day. The slightly lower probability for the fatigued state (P_22_ = 0.86), compared to the vigorous state (P_11_ = 0.92), suggests a modestly higher likelihood of transitioning out of fatigue. While not indicative of substantial volatility, this difference points to a less stable nature of the fatigued state relative to the more persistent vigorous state.

**Figure 4:**
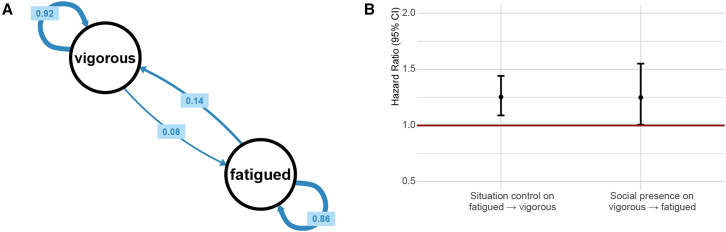
Transition probabilities of the two-state model of vigorous and fatigued states (A) On average, the transition probability at any day to a fatigued state is 8%, in contrast, from fatigued to vigorous state is 14%. (B) Bluetooth devices and situation control both have effects on state transitions, with hazard ratios showing increased likelihood of transitions between states (approximately 1.2–1.4 times more likely) when these factors are present. Parameter values are presented as mean and 95% confidence interval, the red line at 1.0 represents the baseline hazard ratio.

In addition, hazard ratios of covariates for switching states showed a link of external factors (log*N*_BT_, *situation control*) and state transitions. Notably, a higher social presence significantly increased the likelihood of transitioning from vigor to fatigue (hazard ratio, HR = 1.25, 95% CI: 1.01, 1.55), aligning with CTSEM’s indication of associations between social exposure and emotional states. Moreover, *situation control* was as a protective factor, facilitating transitions from fatigue back to vigor (HR = 1.25, 95% CI: 1.09, 1.44). The detailed results are summarized in Table 6 and Figure 4.

**Table 6:** Multi-state model results for vigorous and fatigued states

Parameter	Estimate	95% CI
Mean sojourn time (days)		
State 1 (Vigorous)	10.72	[7.57, 15.17]
State 2 (Fatigued)	6.20	[4.67, 8.22]
Hazard ratios		
Bluetooth devices (log)		
State 1 → State 2	1.25	[1.01, 1.55]
State 2 → State 1	1.00	fixed
Situational control		
State 1 → State 2	1.00	fixed
State 2 → State 1	1.25	[1.09, 1.44]
Transition probabilities (one day)		
State 1 → State 1	0.92	–
State 1 → State 2	0.08	–
State 2 → State 1	0.14	–
State 2 → State 2	0.86	–
Model estimates for vigorous state		
*r*_vigor_	10.46	[9.20, 11.88]
*p*_vigor_	0.44	[0.41, 0.47]
π_vigor_	0.01	[0.01, 0.02]
*r*_fatigue_	1.80	[1.56, 2.09]
*p*_fatigue_	0.25	[0.23, 0.28]
π_fatigue_	0.15	[0.12, 0.17]
*r*_anger_	0.14	[0.07, 0.27]
*p*_anger_	0.16	[0.12, 0.19]
π_anger_	0.20	[0.02, 0.76]
*r*_dejection_	1.26	[0.68, 2.36]
*p*_dejection_	0.48	[0.37, 0.59]
π_dejection_	0.51	[0.41, 0.60]
Model estimates for fatigued state		
*r*_vigor_	7.20	[6.30, 8.23]
*p*_vigor_	0.44	[0.41, 0.47]
π_vigor_	0.01	[0.00, 0.02]
*r*_fatigue_	7.93	[6.96, 9.04]
*p*_fatigue_	0.41	[0.38, 0.44]
π_fatigue_	0.00	[0.00, 0.01]
*r*_anger_	2.02	[1.70, 2.41]
*p*_anger_	0.32	[0.28, 0.35]
π_anger_	0.21	[0.18, 0.24]
*r*_dejection_	2.67	[2.41, 2.95]
*p*_dejection_	0.25	[0.23, 0.27]
π_dejection_	0.00	[0.00, 0.02]

State 1 represents the vigorous state, and state 2 represents the fatigued state. CI = confidence interval. Dashes (−) indicate values not provided in the original data.

## Discussion

In this study, we examined the temporal dynamics between social presence and emotional states using objective Bluetooth measures and three complementary analytical approaches. Our findings revealed a pattern that substantiates the dual nature of social presence—both as momentary source of invigoration and delayed source of accumulated strain.

As predicted in our first hypothesis, social presence was associated with immediate changes in vigor and dejection, and showed relations with delayed increases in fatigue—as in our second hypothesis. This pattern integrates previously disconnected findings in the literature. While research has documented the immediate mood-enhancing effects of social contact^8^^,^^26^ and noted delayed fatigue following social behavior,^18^^,^^27^ our study uniquely demonstrates both effects within the same temporal sequence. The 3–4 h delay in peak fatigue effects aligns remarkably with previous work showing depletion of emotional resources following sustained social interactions after around 3 h.^12^

These findings provide insights relevant to social baseline theory and stress-buffering models, and parallel patterns described in the social allostasis model. Social baseline theory holds that the presence of others serves as humans’ natural operating condition for optimal resource management, potentially boosting mood and vigor by reducing threat and load sharing.^2^ Our results extend this framework by demonstrating that the effects of social exposure may be time-dependent. Increased vigor and reduced dejection associated with contemporaneous social presence support social baseline theory’s core premise about social presence reducing regulatory burden. However, the emergence of delayed fatigue suggests that these induced boosts may have temporal constraints. Similarly, traditional stress-buffering accounts^3^ emphasize how social support protects against stress but do not address the temporal course of these effects specifically. Our continuous-time analyses suggest that while social environments provide immediate stress-buffering benefits, they may prove taxing to emotions over time.

Supporting our third hypothesis, we found a bidirectional relationship where social presence was initially associated with positive mood states but showed relations with increased negative affect over time, while negative mood states were associated with subsequent reductions in social presence. This feedback loop suggests a self-regulatory system where individuals naturally modulate their social exposure based on accumulated emotional costs. Our fourth hypothesis about emotional state stability further refines this finding. While vigorous states showed greater resilience to change compared to fatigued states, this stability became compromised under conditions of higher social presence. Together, these findings reveal a key limitation in both social baseline and stress-buffering theory: while social presence showed initial positive associations with emotional well-being, we observed subsequent relationships with increased fatigue. Interestingly, this pattern is similar to that described in social allostasis theory regarding social interactions.^4^ Our results demonstrate how mere social presence can create patterns of immediate benefits and delayed costs, though it is possible that they are mediated by different pathways than interactive regulatory processes.

To uncover the temporal unfolding and theoretical implications, our study employed several methodological innovations. Crucially, our study employed three complementary analytical approaches to study different aspects of social-emotional dynamics. This multi-method strategy aligns with recent calls for integrative analytical frameworks in psychological research.^28^^,^^29^ LMMs established the foundation by quantifying immediate and lagged associations, while the continuous-time structural equation modeling extended more traditional discrete-time limitations by capturing relationships and bidirectional feedback loops occurring at a fine-grained temporal resolution. In addition, a macro-level perspective by demonstrating how moment-to-moment dynamics translate into broader emotional state transitions and stability patterns were provided by multi-state modeling. By combining these methods, we were not only able to show that the results are robust across analytical approaches but also to assess the unfolding of social-emotional processes across different timescales.

Another methodological strength of our study lies in the robust measurement properties of our emotional state assessments, as demonstrated through generalizability theory analysis. The high generalizability coefficients indicate that our emotional state measurements captured true score variance with high precision. Particularly noteworthy was the variance decomposition, which revealed that for fatigue, vigor, and anger, approximately one-third of the variance could be attributed to stable individual differences, while another third reflected within-person temporal variations. This pattern validates our ability to detect both trait-like stability and meaningful state fluctuations in emotional experiences. These measurement properties strengthen confidence in our observed temporal patterns and provide a solid foundation for the dynamics revealed by our multi-method analytical approach. Beyond these analytical innovations, the convergent validity between Bluetooth-based and self-reported social presence measures—showing nearly identical effect patterns for key emotional states—further strengthens confidence in our approach to capturing real-world social dynamics.

The robust temporal patterns uncovered in our analyses offer valuable insights for real-world social environments. For instance, a possible protective role of perceived situational control indicates that providing individuals greater autonomy over their social environments might help manage regulatory demands. In addition, the 3–4 h delay in fatigue effects suggests the importance of building recovery periods into socially demanding schedules. These findings have important practical implications for managing social interactions in various contexts, such as in work environments, educational settings, and clinical populations—particularly for individuals with depression or social anxiety disorder.

### Limitations of the study

Future studies might benefit from incorporating a broader range of emotional states and interaction types could provide a more nuanced understanding of how different social contexts affect emotional regulation. Also, individual differences in personality traits, particularly extraversion and introversion, likely affect the relationship between social presence and emotional states, suggesting future research should explicitly measure these characteristics to understand their role in social-emotional dynamics. Personality characteristics might specifically influence both the immediate vigor boost and subsequent fatigue patterns we identified.^18^ In addition, the social-emotional dynamics we observed likely operate within broader cultural and environmental contexts that warrant consideration. Cultural norms regarding social interaction and emotional expression may moderate the relationship between social environments and emotional states.^30^ Similarly, environmental settings (e.g., work versus leisure contexts) might influence how social presence affects emotion regulation.^31^ Our study occurred after a period of evolving social norms due to the COVID-19 pandemic, potentially affecting typical patterns of social interaction and their emotional consequences.

In addition, future research could enhance Bluetooth-based social sensing by integrating complementary methodologies. A key limitation of our current approach lies in its binary detection of social presence, which cannot distinguish between different qualities of interpersonal distance (e.g., intimate contact versus distant co-presence) that may have distinct emotional effects. Moreover, while our validation analyses showed significant correlations between Bluetooth device counts and subjectively reported nearby people, several technical constraints should be considered. Bluetooth signals can be detected at significantly greater distances than our 2-meter self-report radius and through walls and floors, potentially inflating presence counts in dense environments, while some individuals may have multiple devices or disable Bluetooth functionality, creating discrepancies between device counts and actual social presence. The absolute difference between self-reported and Bluetooth-detected counts may also reflect that individuals often carry multiple Bluetooth-enabled devices. Consequently, while the correlation between Bluetooth and self-report suggests they capture some shared aspects of the social environment, it’s important to acknowledge that this correlation does not definitely validate either method as a precise measure of social density. Both measures possess inherent limitations, potentially leading to shared inaccuracies, which introduces uncertainty regarding their absolute reliability and validity. Our focus on relative changes mitigates this partly, but the fundamental measurement limitations remain.

Combining Bluetooth proximity data with traditional sociometric badges,^32^ more precise proximity sensors, or smartphone-based interaction logging^10^ could provide richer insights into both the quality and nature of social presence. Such multi-modal sensing approaches could help differentiate between degrees of physical closeness while addressing potential variations in signal detection. This would allow future studies to examine how emotional responses might vary across different types of interpersonal distances, from intimate to public spaces, rather than treating all social presence as equivalent. Moreover, studies incorporating both Bluetooth sensing and physiological measures (e.g., cortisol, heart rate variability) could help distinguish between the biological mechanisms underlying responses to social presence versus those involved in social interaction and allostasis.

In conclusion, by revealing precise temporal dynamics between social presence and emotional states through multiple analytical lenses, our study advances the understanding of how social presence shape well-being across different timescales. These findings support an integrated view of the social environment that both enhance and tax emotional resources, while highlighting promising directions for future research into the mechanisms of socio-emotional dynamics.

## Resource availability

### Lead contact

Requests for further information and resources should be directed to and will be fulfilled by the lead contact, David Willinger (david.willinger@kl.ac.at).

### Materials availability

This study did not generate new materials.

### Data and code availability

• All data have been deposited at the Open Science Framework (https://osf.io/) and are publicly available as of the date of publication. DOIs are listed in the key resources table.
• All original code has been deposited at the Open Science Framework (https://osf.io/) and is publicly available as of the date of publication. DOIs are listed in the key resources table.
• Any additional information required to reanalyze the data reported in this paper is available from the lead contact upon request.

## Acknowledgments

We thank David Lewetz for his technical support. We acknowledge the support by the Open Access Publishing Fund of 10.13039/501100022308Karl Landsteiner University of Health Sciences, Krems, Austria.

## Author contributions

Conceptualization, D.W. and S.S.; methodology, D.W. and S.S.; investigation, S.S. and D.W.; visualization, S.S. and D.W.; project administration,:S.S.; supervision, S.S.; writing—original draft, D.W.; writing—review and editing, S.S. and D.W.

## Declaration of interests

The authors declare no competing interests.

## STAR★Methods

### Key resources table

**Table:** 

REAGENT or RESOURCE	SOURCE	IDENTIFIER
**Deposited data**		

ESM dataset	This paper	Open Science Framework: https://doi.org/10.17605/osf.io/pk3a8

**Software and algorithms**		

RStudio version 2024.04.2	RStudio	https://www.rstudio.com
R version 4.3.3	R Project	https://www.r-project.org
Prolific	Prolific	prolific.com
Scripts for analyses	This paper	Open Science Framework: https://doi.org/10.17605/osf.io/pk3a8

### Experimental model and study participant details

The sample consisted of 59 females and 20 males (*n* = 1 did not respond to demographics questionnaire). Participants’ ages ranged widely, with a mean of 31.5 years (*SD* = 11.2). The average duration of participation was 14.4 days (*SD* = 2.9) with a mean compliance rate of 76.8% (*SD* = 24.9%) corresponding to a mean number of response of *M* = 44.3 (*SD* = 17.4). The sample was further comprised of a mix of singles (*n* = 29), people in relationships (*n* = 31), married or partnered (*n* = 16), and divorced (*n* = 3). Educational backgrounds of the sample was also diverse with comprised university degree (*n* = 36), followed closely by those with high school degree (“Matura” or “Abitur”, *n* = 30), vocational training (*n* = 7) and academy/college (*n* = 4), followed by compulsory education (*n* = 2).

### Method details

#### Power analysis

We conservatively anticipated small effects of *r* = 0.1 between the number of detected Bluetooth devices as a proxy of social interaction and emotional states. We performed a power analysis using *simr*,^33^ which indicated that for 42 repeated assessments (corresponding to a compliance rate of 75% for four assessments per day for two weeks) a minimum sample size of *n* = 21 is required to exceed the power of 80% with alpha level = 5% (using 1,000 simulations, *M* = 84.0%, 95% *CI* [81.6%, 86.2%]). The current study with a sample size of *n* = 80 exceeded this threshold and resulted in a lower bound of the 95% *CI* = 99.6%.

#### Procedure

In this ESM study, we utilized the *ESMira* app, offering robust data collection features including informed consent, encryption, and anonymity.^34^ We recruited participants mainly via the crowd-working platform *Prolific*. Participants had to be at least 18 years, no other inclusion or exclusion criteria were applied. They accessed the study via a project website and QR code, installing *ESMira* on Android or iOS devices. All participants gave their informed consent after they were informed about the goals and possible consequences of the study. After joining, participants completed a brief demographic questionnaire to familiarize themselves with the app's notification system. Data was collected in participants’ natural environments, with *ESMira* prompting four times a day using an acoustic signal. The study encouraged a two-week participation, though participants could withdraw at any time.

To ensure familiarity with the study procedure and app environment, participants could anonymously contact the principal investigator via *ESMira's* chat function at any time during the study. This approach combines technological innovation with participant-centered design, and can facilitate the collection of longitudinal data in the field, eventually enhancing the ecological validity of the collected data. At the end of the study, participants received a reimbursement of up to 16€ based on the overall number of filled-in questionnaire.

The project was conducted in compliance with the declaration of Helsinki and with the local legislation and institutional requirements. The research was exempt from a formal ethics approval (i.e., waiver policy) from the Commission for Scientific Integrity and Ethics of the Karl Landsteiner University of Health Sciences, Austria, as it was non-invasive, anonymous, did not include institutionalized participants (e.g., patients), participation was voluntary, and all participants were 18 years of age or older. The participants provided their written informed consent to participate in this study.

#### Questionnaires

All participants were asked to provide demographic information at the beginning of the study. This included the variables age, sex, relationship status, highest education, and nationality.

In the longitudinal assessment, we used a questionnaire for emotional states (EMOSTAT) consisting of four subscales (vigor, fatigue, dejection, and anger), with each subscale containing 4 distinct items measured on a 4-point Likert scale (1 = *not at all*, 4 = *very*) for a total of 16 items^21^ (Table S1). For all analyses, we calculated the sum score across the items for each of the four subscales. For the *msm* analysis specifically, we shifted the baseline of the sum score to 0 by subtracting 4 for ease of computation. We assessed the reliability of the scale using generalizability theory,^23^ yielding insights into the stability and variability of mood states across the four dimensions.

The Bluetooth scan was implemented in *ESMira* as a separate page in the questionnaire. On this page, participants were informed that with the press of the button displayed on the page, they will start a scan of the environment. Additionally, they were informed that all Bluetooth IDs are pseudonymized, such that the real device ID cannot be traced back. A scan for Bluetooth devices took 60 seconds, in total over 123,000 devices were scanned during the study.

In addition, participants were asked “How many known people are within a visual radius of 2m around you?” and “How many unknown people are within a visual radius of 2m around you?” (underlined in the original text). For this study, we were particularly interested in the total number of people, such that we calculated *n*_people counted_ as the sum of both answers. Lastly, we asked participants about their perceived sense of situational control by asking, “How much do you feel in control of the current situation?”, which they answered on a visual analogue scale (0 = *not at all under control*, 1 = *absolutely under control*).

### Quantification and statistical analysis

#### Validity of Bluetooth measurements

To ensure that Bluetooth-based device proxy accurately reflects the actual people count in the proximate surrounding, we sought to validate the use of Bluetooth measurements using a linear mixed-effects model. The model included random intercepts to account for individual variability per participant.

Note, that the number of Bluetooth devices *n*_BT_ per assessment and reported number of people *n*_people counted_ per time point exhibited significant positive skew (skewness = 6.79 and 4.08, respectively). To address this, we applied a logarithmic transformation of log(*x*+1), which markedly reduced skewness to 0.26 and 1.31, aligning with Bentler's recommendations (±3) for appropriate data transformation.^35^ Thus, the response variable nearby Bluetooth device index was defined as log*N*_BT_ = log(*n*_BT_ + 1), while the predictor variable was log(*n*_people counted_ + 1). The log-transformed variable was also used in subsequent analyses.

#### Reliability of emotional states

To evaluate the measurement reliability of the emotional state questionnaire, we applied generalizability theory using the *R* package *gtheory*.^36^ This approach allowed us to decompose variance components across participants, items, and time points, providing insights into the temporal stability and measurement precision of our emotional state assessments. The generalizability coefficient (ρ^2^) provided an estimate of measurement reliability while accounting for these multiple sources of variance. This analysis was conducted separately for each emotion construct (fatigue, vigor, dejection, and anger) to evaluate their distinct measurement properties.

#### Linear mixed-effects model

Linear mixed-effects models were implemented for analysing contemporaneous and time-lagged associations between Bluetooth contacts and emotional state. We included both, level 1 (within-subject) and level 2 (between-subject) predictors in a joint model using the R package *nlme*.^37^ For this analysis, level 1 predictors were centered around the individual’s mean (centered within cluster, *cwc*) and level 2 predictors (persons’ means) were grand-mean centered (cluster mean centered, *cmc*). This approach allows us to separate within-subject variability from between-subject variability, providing a more precise estimation of the effects. Specifically, it enables us to examine how fluctuations in Bluetooth contacts within an individual over time relate to changes in their emotional state, while also accounting for differences in average emotional state levels between individuals. To account for temporal autocorrelation of data points, we specified the continuous AR(1) correlation structure in the model. For assessing the model fit, we report *R*^2^, specifically addressing how much of the variance in the outcome variable can be explained by the predictors. Two types of *R*^2^ are commonly reported: *R*^2^_marginal_ and conditional *R*^2^_conditional_, each offering distinct insights into the partitioning of explained variance.

*R*^2^_marginal_ quantifies the proportion of variance explained solely by the fixed effects in the model, disregarding any contribution from the random effects. This metric reflects the fit of the fixed predictors across the entire sample.

*R*^2^_conditional_, on the other hand, incorporates both fixed and random effects into the calculation, thereby providing an estimate of the total variance explained by the entire model. This includes not only the fixed predictors but also the random intercepts and slopes, which capture the hierarchical or nested structure of the data. Thus, *R*^2^_conditional_ offers a more comprehensive picture of model fit, reflecting both sample-wide effects and individual-specific variability. We used *t*-tests for assessing the statistical significance of the fixed-effect coefficients, with detailed results reported in the tables.

#### Contemporaneous effects

The contemporaneous effects model examines the relationship between log*N*_BT_ and emotional states at the same time point. The model can be expressed as follows:

##### Assessment-level (level 1)

$$y_{ij}=\beta_{0j}+{\beta_{1j}{EMOSTAT}_{anger}.cwc}_{ij}+{\beta_{2j}{EMOSTAT}_{dejection}.cwc}_{ij}+{\beta_{3j}{EMOSTAT}_{fatigue}.cwc}_{ij}+{\beta_{4j}{EMOSTAT}_{vigor}.cwc}_{ij}+{\beta_{5j}control.cwc}_{ij}+{\left(\beta_{6j}+u_{1j}\right)t}_{ij}+\varepsilon_{ij}$$

##### Person-level (level 2)

$$\beta_{0j}=\gamma_{00}+{\beta_{01}{EMOSTAT}_{anger}.cmc}_{0j}+{\beta_{02}{EMOSTAT}_{dejection}.cmc}_{0j}+{\beta_{03}{EMOSTAT}_{fatigue}.cmc}_{0j}+{\beta_{04}{EMOSTAT}_{vigor}.cmc}_{0j}+{\beta_{05}control.cmc}_{0j}+u_{0j}$$

where

$$\varepsilon_{ij}\;∼\;N\left(0,\sigma^{2}\right)$$

$$u_{0j}\;∼\;N\left(0,\sigma_{u_{0}}^{2}\right)$$

$$u_{1j}\;∼\;N\left(0,\sigma_{u_{1}}^{2}\right)$$

$$cor\left(\varepsilon_{ij},\varepsilon_{\left(i+1\right)j}\right)=\rho^{\left|t_{ij}-t_{\left(i+1\right)j}\right|}$$

and $\gamma_{00}$ is the grand mean of participants, indexed by *j*, and measurements, indexed by *i*, $u_{0j}$ is the deviation from the grand mean, $\varepsilon_{ij}$ represents the residual, $y_{ti}$ is the log*N*_BT_, ρ is the coefficient of the temporal autocorrelation, and *t* represents the continuous time in days from the individual start of the study.

#### Lagged effects

Similarly, to assess time-lagged effects, we created predictors that were shifted in time by one assessment (*i*-1). This can be expressed as:

##### Assessment-level (level 1)

$$y_{ij}=\beta_{0j}+{\beta_{1j}{EMOSTAT}_{anger}.cwc}_{\left(i-1\right)j}+{\beta_{2j}EMO{STAT}_{dejection}.cwc}_{\left(i-1\right)j\;}+{\beta_{3j}{EMOSTAT}_{fatigue}.cwc}_{\left(i-1\right)j}+{\beta_{4j}EMO{STAT}_{vigor}.cwc}_{\left(i-1\right)j}+{\beta_{5j}control.cwc}_{\left(i-1\right)j}+{\left(\beta_{6j}+u_{1j}\right)t}_{\left(i-1\right)j}+\varepsilon_{ij}$$

Model checks were performed using the *performance* package in *R*.^38^ The checks for multi-collinearity indicated no particular problem for the predictors (contemporaneous: variance inflation factor, VIF < 3.2; lagged: VIF < 3.5). There was an indication for both models of heteroscedasticity (*p* < .001). Note, however, that for this sample size these checks tend to be significant even for small deviations.

#### Continuous-time structural equation model

We extended the discrete-time analysis with LMM using continuous-time structural equation modeling (*ctsem* in attempt to better capture the temporal dynamics between latent factors of negative mood state and the Bluetooth device count log*N*_BT_. This approach allows for more precise modeling of continuously fluctuating processes and their interactions, overcoming limitations of discrete-time models.^24^

At the core of *ctsem* is the stochastic differential equation typically noted as:

$$\begin{array}{c} d\eta \left(t\right)=\left[A\eta \left(t\right)+b+M\chi \left(t\right)\right]dt+GdW\left(t\right) \end{array}$$

Here, η(t) represents the latent state vector, A is the drift matrix capturing temporal effects, b accounts for time-dependent covariates (e.g., *situation control*), *G* is the diffusion matrix, and d*W*(*t*) represents Gaussian white noise, with the two latter comprising the “system noise” *GdW*(*t*).

The measurement model links the latent factors to the observed variables:

$$\begin{array}{c} y\left(t\right)=\Lambda \eta \left(t\right)+\tau +\varepsilon \left(t\right),\varepsilon \left(t\right)\;∼\;N\left(0_{c},\Theta \right) \end{array}$$

where *y*(*t*) is the vector of observed variables, Λ contains factor loadings, τ represents intercepts, and ε(t) captures the measurement error with covariance matrix Θ.

We modeled negative mood state using observed variables of *fatigue*, *vigor*, *dejection*, and *anger* from the emotional state questionnaire. Social presence was operationalized through log*N*_BT_. Factor loadings for emotional state items were estimated, constraining vigor to have a negative loading to ensure construct validity.

The parameters of the drift matrix and diffusion matrix were estimated to capture the dynamic interplay between latent factors. Notably, we allowed off-diagonal elements of *A* to vary between participants, accommodating individual differences in cross-factor influences. This approach allows to investigate of how negative mood states and social proximity co-evolve over time, while accounting for situation-specific influences through the inclusion of the self-report variable *situation control* as a time-dependent covariate.

#### Markov multi-state model

To complement the *ctsem* framework, we implemented a two-state latent Markov multistate model^39^ to specifically explore the transition dynamics between distinct mood states (e.g., fatigued and vigorous), offering a key advantage in capturing transitions which *ctsem* does not. By focusing on categorical shifts between latent mood states—defined by the emotion constructs in the emotion state questionnaire—the model allows to (1) uncover any meaningful latent emotional states and (2) a direct analysis of factors influencing these transitions. Additionally, the multistate model enables the investigation of each state’s emotional profile by examining the distribution of observed emotional responses within each latent state, offering insights into the specific characteristics of each mood state. The core equation for modeling transition intensities between latent states is:

$$\begin{array}{c} q_{ij}\left(t\right)=\alpha_{ij}\;\exp\left[\beta^{T}z\left(t\right)\right] \end{array}$$

where $q_{ij}\left(t\right)$ denotes the transition intensity from state i to state j at time t, $\alpha_{ij}$ represents the baseline transition intensity, β is a vector of coefficients, and z(t) is a vector of time-dependent covariates.

In our study, the variables log*N*_BT_ and *situation control* variables were included as time-dependent covariates, influencing the transition probabilities. This allowed us to capture the external influences on emotional state transitions. Following an inspection of the emotional states distributions, we implemented a zero-inflated negative binomial model for the observation model, using a Bernoulli process to account for the generation of zeros. This decision was driven by the high frequency of zero values and overdispersion observed in the data. Thus, the observation model followed the following probability mass:

$$P\left(Y=y\right)=\left\{ \begin{array}{c} \pi +\left(1-\pi \right)\left(\frac{\Gamma \left(y+r\right)}{y!\Gamma \left(r\right)}{\left(1-p\right)}^{r}p^{y}\right)\;if\;y=0\\ \left(1-\pi \right)\left(\frac{\Gamma \left(y+r\right)}{y!\Gamma \left(r\right)}{\left(1-p\right)}^{r}p^{y}\right)\;if\;y>0 \end{array}\right.$$

with π corresponding to the probability of observing an excess zero, and r and p are the shape and success probability parameters of the negative binomial distribution, respectively.
