# Supplementary material for: Bluetooth-sensed social presence is associated with immediate vigor and delayed fatigue: A multi-method time series analysis
Source: iScience. 2025 May 22;28(6):112726. doi: 10.1016/j.isci.2025.112726 (PMC12179627; doi:10.1016/j.isci.2025.112726)

iScience, Volume *28*

Supplemental information

Bluetooth-sensed social presence is associated with immediate vigor and delayed fatigue: A multi-method time series analysis

David Willinger and Stefan Stieger

Supplementary Table 1. Emotion state questionnaire, related to Table 1 and 2

Item	Mean	SD
Dejection		
Discouraged (entmutigt)	2.06	1.18
Hopeless (hoffnungslos)	2.04	1.31
Blue (schwermütig)	2.06	1.26
Uncertain about things (unsicher)	2.24	1.23
Vigor		
Energetic (schwungvoll)	3.39	1.04
Vigorous (tatkräftig)	3.73	0.96
Alert (munter)	3.95	0.98
Lively (lebhaft)	3.71	1.03
Fatigue		
Exhausted (erschöpft)	3.17	1.20
Weary (müde)	3.41	1.06
Listless (abgeschlafft)	2.91	1.22
Worn out (ermattet)	2.91	1.25
Anger		
Angry (zornig)	1.32	0.46
Furious (wütend)	1.36	0.48
On edge (gereizt)	1.77	0.78
Annoyed (verärgert)	1.55	0.53

Note. German translations of items used in the study are reported in brackets.

Supplementary Table 2. Variance decomposition of the emotion state questionnaire, related to Method section “Reliability of emotional states”

Item	Fatigue		Vigor		Dejection		Anger	
	σ^2	**%**	σ^2	**%**	σ^2	**%**	σ^2	**%**
Time:Person	0.94	32.5	0.86	34.7	0.35	15.1	0.35	36.1
Item:Person	0.14	4.7	0.16	6.5	0.13	5.7	0.05	5.5
Time:Item	0.00	0.1	0.00	0.00	0.00	0.00	0.00	0.1
Person	1.14	39.7	0.80	32.4	1.40	60.5	0.26	27.1
Time	0.01	0.5	0.00	0.2	0.01	0.2	0.00	0.2
Item	0.05	1.8	0.05	2.1	0.01	0.3	0.04	3.9
Residual	0.60	20.7	0.60	24.2	0.42	18.1	0.26	27.0

Supplementary Figure 1. Distribution and relationship of Bluetooth device counts and reported nearby people, related to Result section “Sample characteristics”. (Top) Histograms showing the frequency distributions of Bluetooth device detections (left) and self-reported counts of people within 2m distance (right). Both measures display right-skewed distributions with the majority of observations at lower values. (Bottom) Scatterplot illustrating the relationship between the number of detected Bluetooth devices and self-reported people counts, with a linear regression line. The positive correlation suggests Bluetooth device counts serve as a reasonable proxy for social presence.

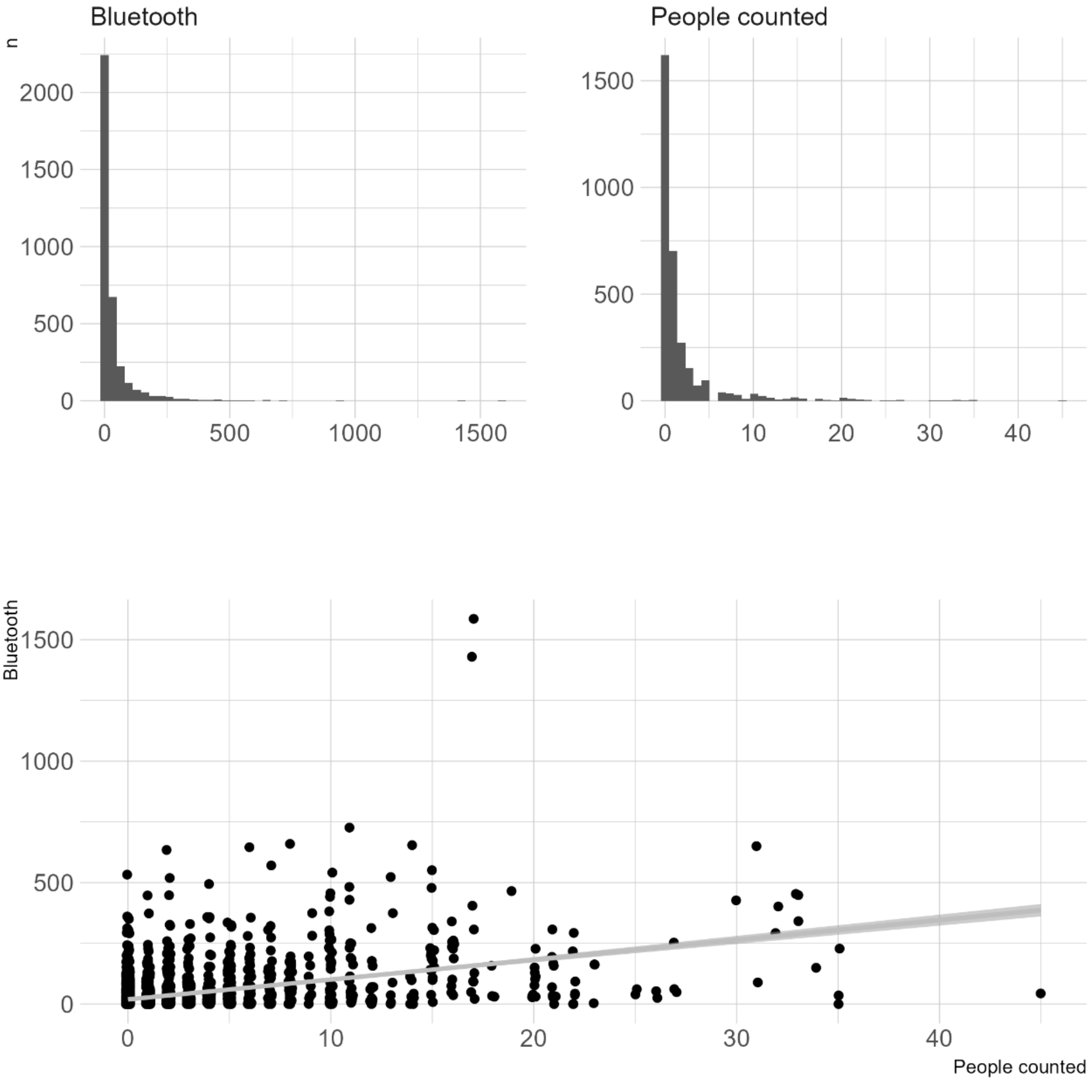

Supplement: Document S1. Figure S1 and Tables S1 and S2 [file mmc1.pdf]
